# Integrative analysis of long noncoding RNA and mRNA reveals candidate lncRNAs responsible for meat quality at different physiological stages in Gushi chicken

**DOI:** 10.1371/journal.pone.0215006

**Published:** 2019-04-09

**Authors:** Donghua Li, Fang Li, Keren Jiang, Meng Zhang, Ruili Han, Ruirui Jiang, Zhuanjian Li, Yadong Tian, Fengbin Yan, Xiangtao Kang, Guirong Sun

**Affiliations:** 1 College of Animal Science and Veterinary Medicine, Henan Agricultural University, Zhengzhou, China; 2 Henan Innovative Engineering Research Center of Poultry Germplasm Resource, Zhengzhou, China; University of Illinois, UNITED STATES

## Abstract

Long noncoding RNAs (lncRNAs) play important roles in transcriptional and posttranscriptional regulation. However, the effects of lncRNAs on the meat quality of chicken hasn’t been elucidated clearly yet. Gushi chickens are popular in China because of their superior meat quality, particularly the tender flesh, and unique flavor. Gushi chickens are popular in China because of their superior meat quality, delicate flesh, and unique flavor. We performed RNA-Seq analysis of breast muscle from Gushi chicken at two physiological stages, including juvenile (G20W) and laying (G55W). In total, 186 lncRNAs and 881 mRNAs were differentially expressed between G20W and G55W (fold change ≥ 2.0, *P* < 0.05). Among them, 131 lncRNAs presented upregulated and 55 were downregulated. We identified the *cis* and *trans* target genes of the differentially expressed lncRNAs, and constructed lncRNA-mRNA interaction networks. The results showed that differentially expressed mRNAs and lncRNAs were mainly involved in ECM-receptor interaction, glycerophospholipid metabolism, ubiquitin-mediated proteolysis, and the biosynthesis of amino acids. In summary, our study utilized RNA-seq analysis to predict the functions of lncRNA on chicken meat quality. Furthermore, comprehensive analysis identified lncRNAs and their target genes, which may contribute to a better understanding of the molecular mechanisms underlying in poultry meat quality and provide a theoretical basis for further research.

## Introduction

Along with the improvement in life quality in modern society, more and more consumers paid attention to the food safety and food quality, such as the meat quality, which includes color, flavor, juiciness, fat content and tenderness. The market demands guided the direction of breeding program in poultry industry, which has made a conversion to balance the growth rate and meat quality to some extent. Meat quality is significantly affected by a series of complex factors, including breed, age, genetics, nutrition and feeding environment [1–5]. The Gushi chicken was an eminent representative with high meat quality of indigenous chicken breeds, which origins from the Gushi county of Henan province in China. The high meat quality performs in many facets. For example, high intramuscular fat (IMF) content, low shear force (SF), low drip loss, low pH decreasing, nice color and flavor. In our previous study, the laying-stage Gushi hens (55 weeks, G55W) exhibited higher serum lipid levels, IMF deposition capacity, water holding capacity (WHC), SF, intermuscular fat width (IFW) and unsaturated fatty acid (UFA) levels than the juvenile Gushi hens (20 weeks, G20W) showed, while G20W showed higher drip losses and muscle tenderness. Global DNA methylation profiles indicated that G55W exhibited higher global DNA methylation levels than G20W [2]. But phenotypic characteristics of meat quality are not only controlled by coding genes but also by noncoding genes at transcriptional regulatory level. Although many studies have investigated meat quality differences among different breeds at the mRNA level [6–8], little has been demonstrated between the different physiological stages from the same breed.

Long noncoding RNAs (lncRNAs), or mRNA-like long noncoding RNAs, are both important members of the noncoding RNA families, with lengths ranging from 200 bp to > 100 kb [9–13]. In the past, lncRNAs were thought to have no coding ability due to their lack of typical open reading frames, but recent studies have found that lncRNAs have small open reading frames (sORF) that could encode small peptides and these small peptides have important biological functions [14–16]. These studies have expanded the traditional understanding of the potential for genetic coding within the genome and have elucidated the rich variety and diversity of lncRNAs. Recent studies have shown that lncRNAs play specific roles in the development of different organs and tissues. For example, the lncRNA Bvht can affect the development of cardiac tissue in mice, as the RNAi knockout of Bvht can change the expression of cardiac specific genes, and inhibit the normal development of neonatal cardiomyocytes into mature cardiomyocytes [17]. Linc-MD1, Lnc-mg and Linc-RAM were observed to be involved in myogenesis [18–20]. LncRNA-Six1 could promote the cell proliferation, which participated in muscle growth [21]. However, little is known what role of lncRNAs played in regulating the meat quality of breast muscle from chickens at different physiological stages.

Herein, we conducted a comprehensive transcriptome profile on breast muscle tissue in juvenile and laying-stage Gushi hens. The results revealed the expression patterns of mRNAs and lncRNAs, which were important during different developmental stages in the two groups. An integrated analysis of differentially expressed (DE) lncRNAs and mRNAs was performed to elucidate the regulatory patterns of lncRNAs and their interactive network with putative target genes. Our study will help to identify the predicted interplay between lncRNAs and protein-coding genes in breast muscle, and the information generated from these predictions can be utilized in further studies of lncRNA function in chicken breast meat quality. This study may also provide insights into gene regulation and lay a foundation for genetic breeding strategies to improve meat quality.

## Materials and methods

### Experimental animals and sample collection

All animal experiments were performed in accordance with a protocol approved by the Institutional Animal Care and Use Committee (IACUC) of Henan Agricultural University [22]. The animals involved in this study were humanely sacrificed as necessary to ameliorate suffering.

Animals were raised in the same environment with the same ad libitum diet. Six healthy Gushi hens were selected randomly at 20 weeks old (G20W) and 55 weeks old (G55W), representing juvenile and laying-stage hens, respectively. Three individuals per stage were regarded as biological replicates. Samples were harvested from the breast muscle, snap frozen in liquid nitrogen and subsequently stored at -80°C.

### RNA extraction and Solexa sequencing

Total RNA was extracted from chicken breast tissues using TRIzol reagent (Invitrogen, USA) following the manufacturer’s protocol. RNA quality was determined using the RNA Nano6000 Assay Kit of the Bioanalyzer 2100 system (Agilent Technologies, CA, USA), which analyzes the integrity of the RNA. The cDNA libraries for RNA-Seq were constructed using a TruSeq RNA Sample Preparation Kit (San Diego, CA, USA) according to its instructions. First, purification of mRNA was isolated by polyA selection with magnetic oligo (dT) beads, and the purified products were randomly sheared in Fragmentation Buffer. Second, cDNA was synthesized with random primers. Then, the double-stranded cDNA was end-repaired, A-bases were added, and adaptors were ligated, and the ligation products were purified. At last, cDNA libraries were obtained by PCR enrichment. Finally, six libraries were sequenced at Novogene Co. Ltd. (Beijing, China) on an Illumina HiSeq 2000 with 2×150 paired-end (PE) reads. The RNA-Seq dataset supporting the conclusions of this article is available in the Sequence Read Archive (SRA) at the National Center for Biotechnology Information (NCBI) under accession numbers SRR5367021, SRR5367022, SRR5367023, SRR5367024, SRR5367025 and SRR5367026.

### Data analysis

After sequencing, the raw data were stored in fastq format (http://en.wikipedia.org/wiki/Fastq). First, clean data were obtained by removing the low-quality reads, reads containing an unknown nucleotide “N” and duplicate sequences and trimming the first 15 nt at the5’-end. The Q20, Q30 and GC content of the clean data were calculated [23]. The resulting clean, high-quality data were used for all downstream analyses. The clean, paired-end reads were aligned to the chicken genome sequence assembly using TopHat [24], and the mapped reads for each sample were assembled using both Scripture (beta2) [25] and Cufflinks [26].

### LncRNA identification and prediction of target genes

To ensure the quality of the obtained lncRNAs, four criteria were used to identify the desired lncRNAs in the transcriptome assemblies: 1) transcripts with length ≤ 200 nt, exon number ≤ 2 and open reading frame (ORF) length ≥300 nt were removed; 2) Cufflinks was used to calculate the read coverage of every transcript [27], and transcripts with an FPKM value of more than 0.01 were removed; 3) the remaining transcripts were BLASTed against known chicken lncRNAs in ALDB, and only lncRNA transcripts, for which the splice sites were completely congruent between our results and those in ALDB, were immediately labeled in our results as known lncRNAs; and 4) the coding potential of the transcripts was predicted using the coding potential calculator (CPC < 1) [28], Coding-Potential Assessment Tool (CPAT) [29], Coding Noncoding Index (CNCI) [30], and Pfam [31] protein domain families to further remove coding genes, Subsequent steps were performed based on the intersection of the results obtained using the four databases. Gene transcripts within 100 kb upstream or downstream of the lncRNA were considered *cis* target genes. The correlation between lncRNA and target genes was analyzed using Pearson’s correlation coefficient. The lncRNA-gene pairs with absolute values of the coefficients greater than 0.95 were retained.

### GO and KEGG analysis

Gene Ontology (GO) enrichment analysis and Kyoto Encyclopedia of Genes and Genomes (KEGG) pathway analysis were performed assessed using DAVID (http://david.abcc.ncifcrf.gov/). We retained the “GO terms” and “KEGG pathways” with *P*-values < 0.05.

### LncRNA-mRNA network analysis

Pearson’s correlation was used to determine whether the expression levels of DE lncRNAs were correlated with DE mRNAs between G20W and G55W. The lncRNA-mRNA correlations with *P*-values <0.05 were retained. We used Cytoscape 3.4.0 to visualize lncRNA-mRNA networks.

### Quantitative RT-PCR and data analysis

To verify the accuracy of the data obtained by high-throughput sequencing, nine lncRNAs were randomly selected, and the results were confirmed by qRT-PCR for nine randomly selected lncRNAs. Total RNA was isolated from six Gushi chicken breast muscle samples at 20weeks and 55weeks of age, which was the same as for the RNA-seq samples. All gene-specific qRT-PCR primers were designed using Primer 6.0 software and synthesized by Shenggong Biological Engineering (Wuhan) Limited by Share Ltd. All qPCR was conducted using the LightCycler 96 Real-Time PCR system (Roche Applied Science). In a 10 μL reaction mixture, 1.0 μL of cDNA was used for amplification with 5 μL of SYBR Premix Ex Taq (TRNaseH Plus) (TaKaRa, Dalian, China), 0.5 μL of forward primer (10 μM), 0.5 μL of universal primer (10 μM), and 3 μL of deionized water. The reactions were incubated at 95°C for 1 min, followed by 35 cycles of 95°C for 15 s, 60°C for 45 s, and 72°C for 40 s and an extension for 10 min at 72°C. All reactions were performed in triplicate. The threshold cycle (CT) was determined using the default threshold settings, and the data were analyzed using the 2^–ΔΔCt^ program [32]. Statistical analyses of the data were performed using SPSS 22.0 (SPSS Inc., Chicago, IL, USA). The data are presented as the means ± standard error (SE).

## Results

### Overview of lncRNA expression

**Fig 1:**
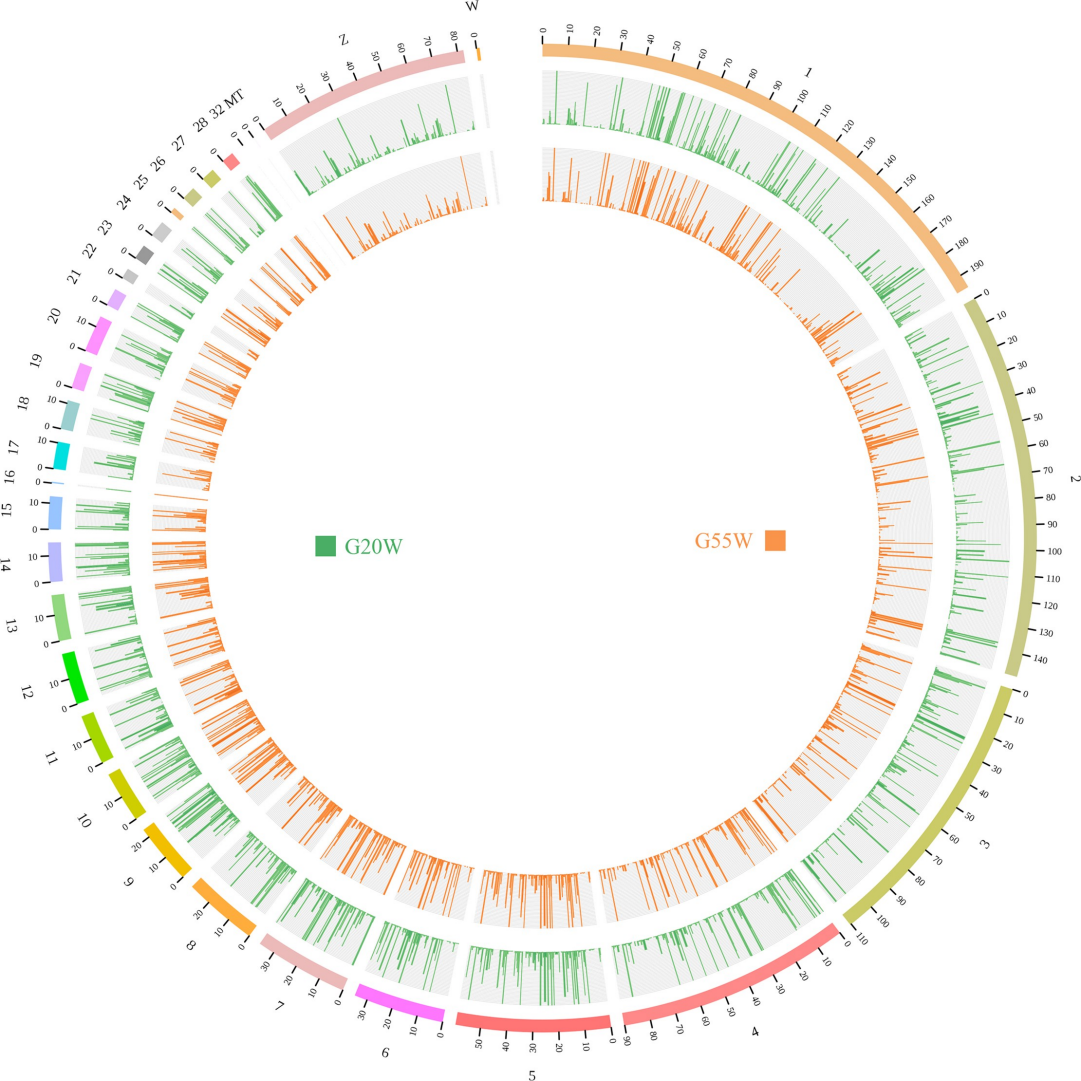
Distribution of identified lncRNAs on each chromosome. The outer ring represents the chicken genome labeled with chromosome number and position. The green circle shows the distribution of identified lncRNAs in G20W, and the orange circle shows the distribution in G55W.

To identify DE lncRNAs, we established six cDNA libraries that represented two different physiological stages: G20W-1, G20W-2, and G20W-3 from the breast muscle tissue of 20-week-old juvenile hens and G55W-1, G55W-2, and G55W-3 from the breast muscle tissue of 55-week-old laying hens. RNA sequencing obtained a total of 43.51 Gb of data, each stage averaging 7.3 Gb of data. More than 61.05% of the clean reads were perfectly mapped to the chicken reference genome, and 59.69 to 63.33% uniquely mapped reads were obtained from the total reads from the six samples. The Q30 results in each sample were > 90%, and the GC percentage was less than 60%, as listed in Table 1. After mapping the reads to each chromosome, no significant difference between the different physiological stages was observed, and the number of reads mapping to different chromosomes was consistent with the chromosome length (Fig 1). The same results were obtained in a previous study [33].

**Table 1:** Quality control results and high-quality clean reads obtained for each sample.

Sample name	Raw reads	Clean reads	Clean bases	Q30(%)	GC content(%)	Total mapped	Uniquely mapped
G20w_1	61074958	58371820	8.76G	91.47	54.07	37449099 (64.16%)	36581329 (62.67%)
G20w_2	43792298	41295976	6.19G	90.17	54.36	25210155 (61.05%)	24648492 (59.69%)
G20w_3	48559334	46058428	6.91G	90.36	53.2	29827714 (64.76%)	29167154 (63.33%)
G55w_1	53380896	50633158	7.59G	90.35	53.34	32080591 (63.36%)	31377023 (61.97%)
G55w_2	47745344	45647898	6.85G	90.8	53.72	29144264 (63.85%)	28499986 (62.43%)
G55w_3	50690090	48051564	7.21G	90.54	53.68	30166068 (62.78%)	29414761 (61.21%)

**Fig 2:**
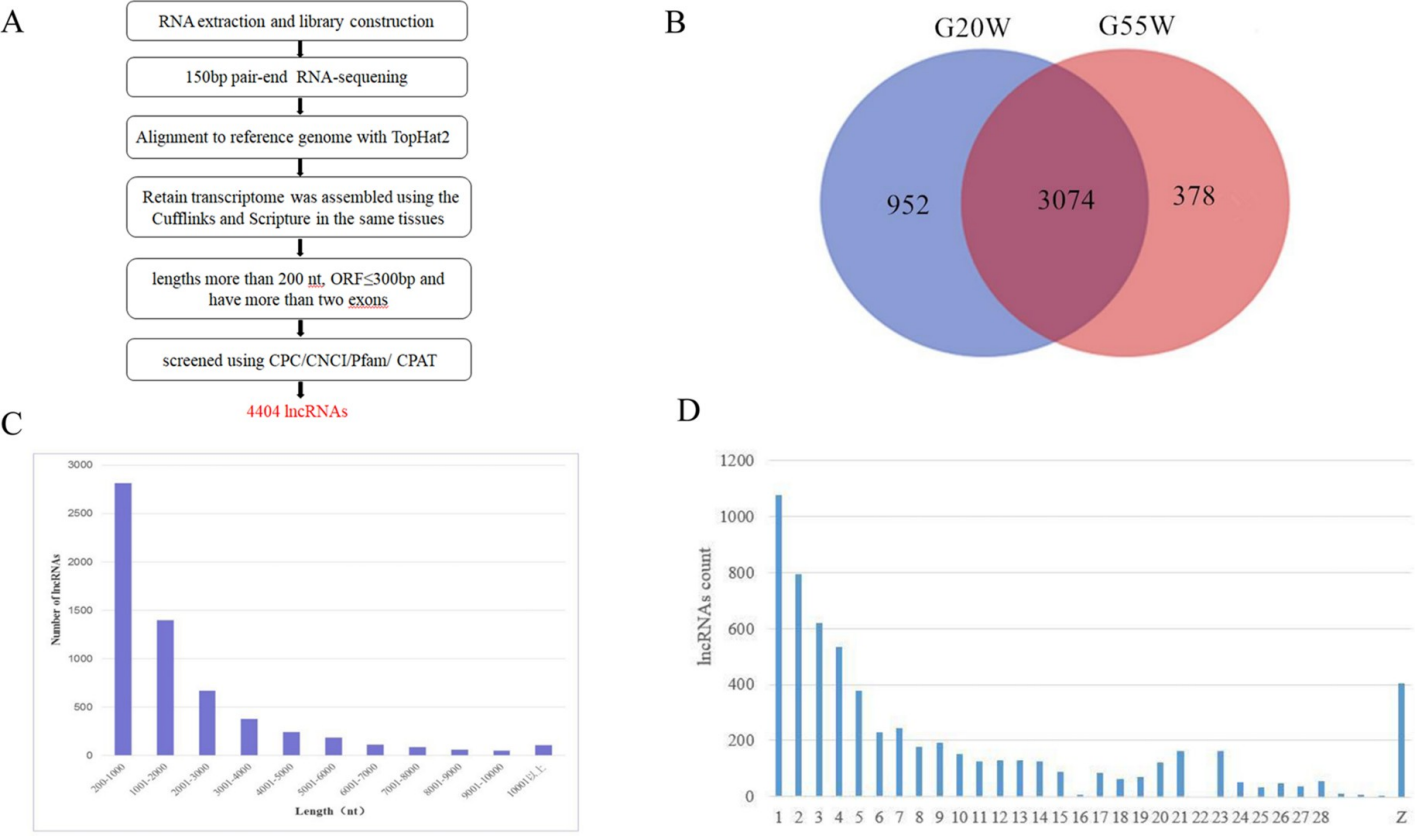
Characteristics of lncRNAs. (A) Computational pipeline for the systematic identification of lncRNAs. (B) Venn diagram illustrating the overlap of lncRNAs in breast muscle tissue libraries from G20W and G55W chickens. G20W: 20 weeks of age; G55W: 55 weeks of age. Length (C) and chromosomal (D) distribution of Gushi chicken lncRNAs.

After assembly, we obtained 4, 404 lncRNAs from the two groups (Fig 2A). Among the total lncRNAs, 4, 029 and 3, 452 lncRNAs were identified in the G20W and G55W samples, respectively, with 3, 074 lncRNAs observed to be expressed in both groups. We found that 952 lncRNAs were specifically expressed in G20W and that 378 lncRNAs were expressed only in G55W (Fig 2B). Furthermore, we examined the lengths and chromosomal locations of the lncRNAs. The length of the lncRNAs ranged from 200 to 10, 000 nucleotides (nt). 60% of the total number of lncRNAs were approximately 200 to 2, 000 nt in length (Fig 2C). Interestingly, the lncRNAs in chicken breast tissue were similar in length to the lncRNAs in mouse and Atlantic salmon [34, 35]. To understand the distribution of the chicken lncRNA reference genome, we analyzed the distribution of lncRNAs in Gushi chicken breast samples. The results showed that lncRNAs were distributed across the chicken chromosomes (chromosomes 1–28, W), with chromosomes 1–4 and Z containing many lncRNAs (Fig 2D). that is, the larger chromosomes contained more lncRNAs. Widespread transcription along all chromosomes with some bias in transcriptional activity on specific chromosomes indicates that the presence of lncRNAs is not due to transcriptional noise.

### Differentially expressed lncRNAs and mRNAs in chicken breast muscle

**Fig 3:**
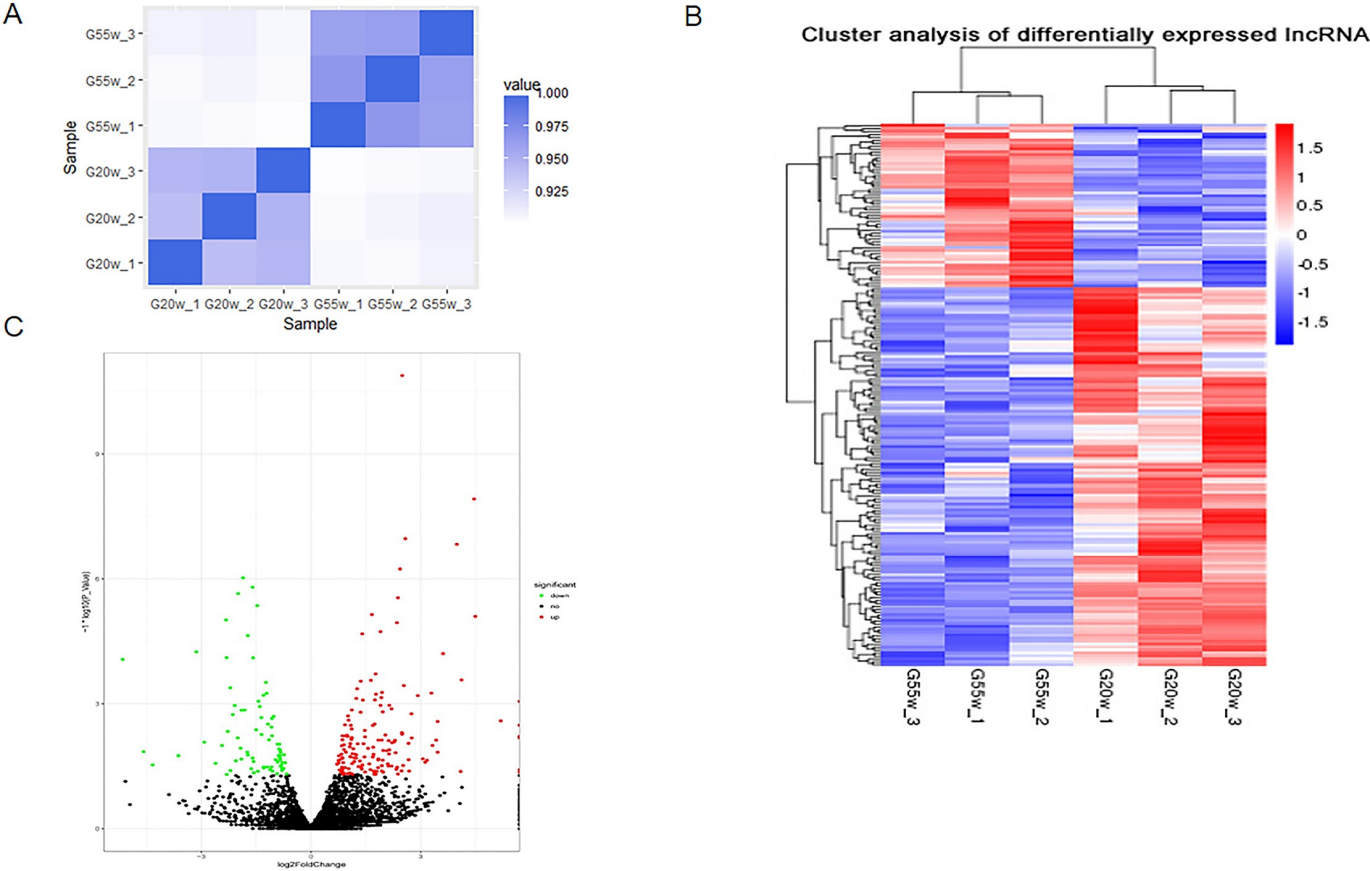
Analysis of differentially expressed lncRNAs. (A) Representative of the degree of similarity between samples. The correlation coefficient is represented by color; deeper color represents a stronger correlation. (B) Clustering analysis of differentially expressed lncRNAs in G20W and G55W. (C) Volcano plot of differentially expressed lncRNAs in G20W and G55W. Green and red represent downregulated and upregulated expression, respectively.

The correlation coefficient can represent the degree of similarity between samples. We found that G20W and G55W were highly correlated (Fig 3A). We used Heatmap3 [36] to perform an unsupervised cluster analysis of the differences in lncRNA expression in breast muscle tissue at different developmental stages. The results of the cluster analysis showed that distinct lncRNA expression patterns are associated with G20W and G55W (Fig 3B). We identified 234 DE lncRNAs between the G20W and G55W breast muscle tissues (|Log2 Fold-Change| ≥ 1.0, *P* < 0.05). Among the total 234 lncRNAs, 131 were upregulated in G20W compared with G55W, and 55 were downregulated (Fig 3C). Among the top 10 expressed lncRNAs in the libraries, the first 7 lncRNAs were present in both samples. None of the top 10 lncRNAs were specifically expressed in G20W or G55W. The fold change in the expression of the DE lncRNAs varied from -1.8582 to 2.3798 (Table 2). These findings may help to identify DE lncRNAs at specific stages in chickens.

**Table 2:** The top 10 most abundantly expressed lncRNAs in breast muscle from G20W and G55W chickens.

Rank in G20W vs G55W	Gene ID	Readcount G20W	Readcount G55W	log2 fold change	*P-*value
1st-3rd	ALDBGALG0000005167	2701.139661	5610.348	-1.0545	0.041376
2nd-5th	ALDBGALG0000001104	1460.518618	4047.131	-1.4704	4.36E-06
3rd-7th	ALDBGALG0000002353	927.104675	2803.853	-1.5966	1.58E-06
4th-1th	ALDBGALG0000005114	13872.07242	2791.343	2.3132	0.014968
5th-10th	ALDBGALG0000000119	809.0155205	2677.644	-1.7267	2.29E-05
6th-9th	ALDBGALG0000001999	868.9814675	1769.198	-1.0257	0.00199
7th-2th	ALDBGALG0000004424	7212.919404	1385.895	2.3798	2.84E-06
8th-14th	ALDBGALG0000000747	518.9016945	1270.762	-1.2922	0.03456
9th-25th	ALDBGALG0000005602	179.7971973	651.8531	-1.8582	9.42E-07
10th-17th	ALDBGALG0000003588	268.1563711	630.4646	-1.2333	0.000305
11th-4th	ALDBGALG0000000333	2227.430031	586.7292	1.9246	0.000707
13th-6th	ALDBGALG0000002811	1065.111742	491.5267	1.1157	0.001404
15th-8th	ALDBGALG0000001219	884.7245861	334.9795	1.4012	2.08E-05

To further compare transcriptional profiles and networks in breast tissue, gene expression profiling was performed using the Illumina HiSeq 2000. Finally, we identified a total of 881 DE genes in breast tissue samples, comprising 451 upregulated and 430 downregulated genes in G20W compared with G55W (|Log2 fold-change| ≥ 1.0, *P* < 0.05).

### GO enrichment analysis

We predicted the *cis* and *trans* targets of DE lncRNAs to further investigate how lncRNAs interact with the mRNA of their target genes to regulate chicken breast meat quality and to identify key molecular participants in this process. LncRNA can not only regulate the expression of neighboring protein-coding genes through a *cis* mechanism [37, 38], but also regulate the expression of genes located on other chromosomes via a *trans* mechanism [21, 39]. In this study, bioinformatic analysis was used to predict the potential target genes of DE lncRNAs via *cis* and *trans*. A total of 315 *cis*-mRNA pairs (S1 Table) and 15, 722 random *trans*-mRNA pairs (S2 Table) were identified in the present study. Furthermore, we identified the genes common to both categories and were therefore able to filter key lncRNAs and their associated target genes that could affect the quality of breast muscle tissue.

**Fig 4:**
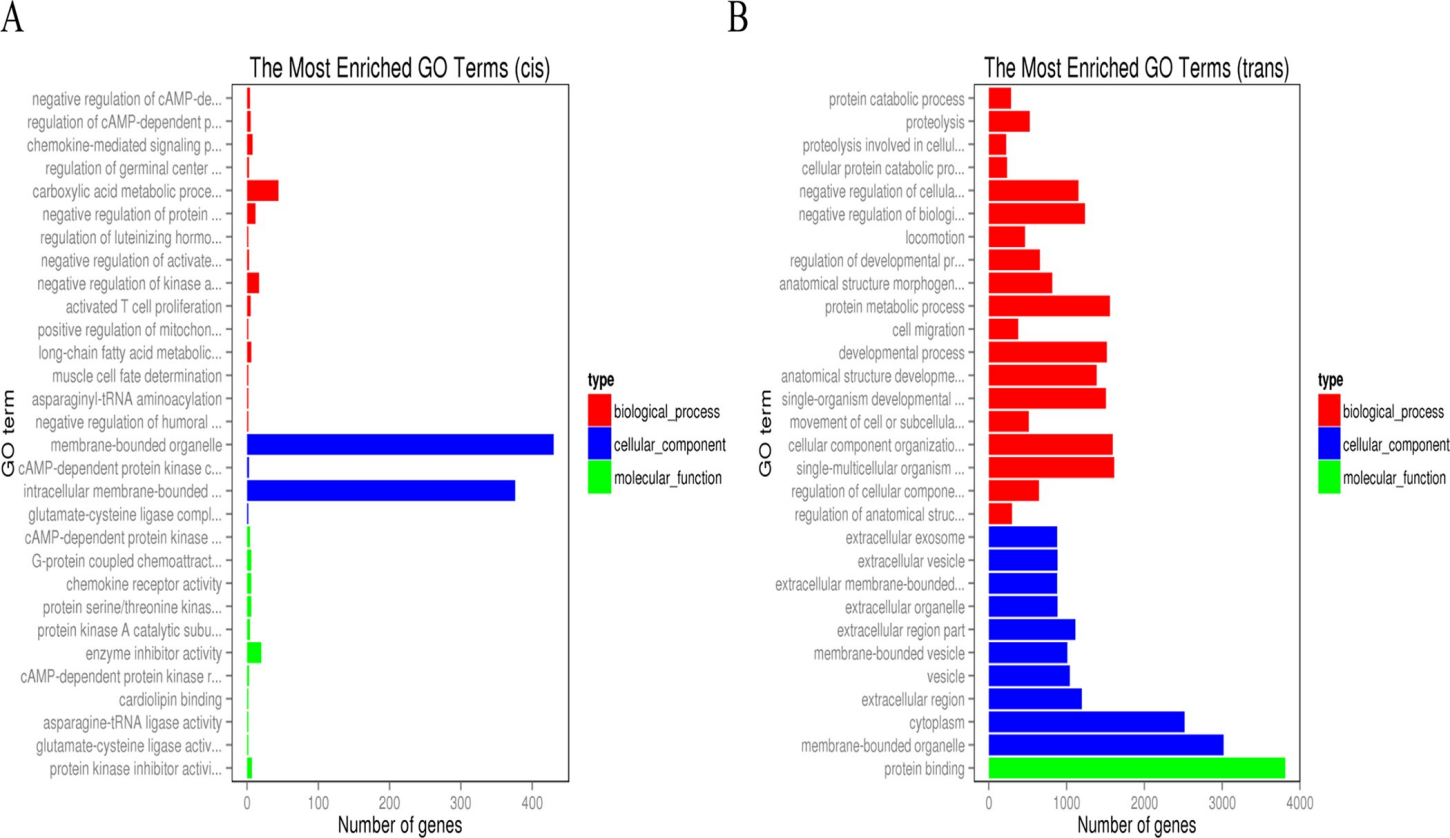
GO analyses of differentially expressed lncRNAs and mRNAs. (A) Categories of biological processes, cellular components and molecular functions of the target genes of differentially expressed lncRNAs and mRNAs (*cis*). (B) Categories of the biological processes, cellular components and molecular functions of the target genes of differentially expressed lncRNAs and mRNAs (*trans*).

We used GOseq to compare the GO classifications of the groups of upregulated and downregulated genes (adjusted *P* < 0.05) [40]. Overall, 203 DE lncRNAs and coexpressed mRNAs were enriched for 955 GO terms in the *cis* targets of lncRNAs. Among them, 689 of the enriched GO terms were categorized as biological processes (BP), 153 as for molecular functions (MF), and 113 ascellular components (CC). The top 30 significantly enriched GO terms are shown in Fig 4A. The most significantly enriched GO term between the G20W and G55W groups was cAMP-dependent protein kinase inhibitor activity. For *trans* targets, 774 DE lncRNAs and coexpressed mRNAs were enriched for 6, 398 GO terms. Among them, 4, 852 GO terms were enriched for BP, 961 were enriched for MF, and 585 were enriched for CC. The top 30 significantly enriched GO terms are shown in Fig 4B. The top three GO terms were membrane-bound protein binding, protein metabolic process, and cellular component organization or biogenesis.

### KEGG pathway enrichment analysis

**Fig 5:**
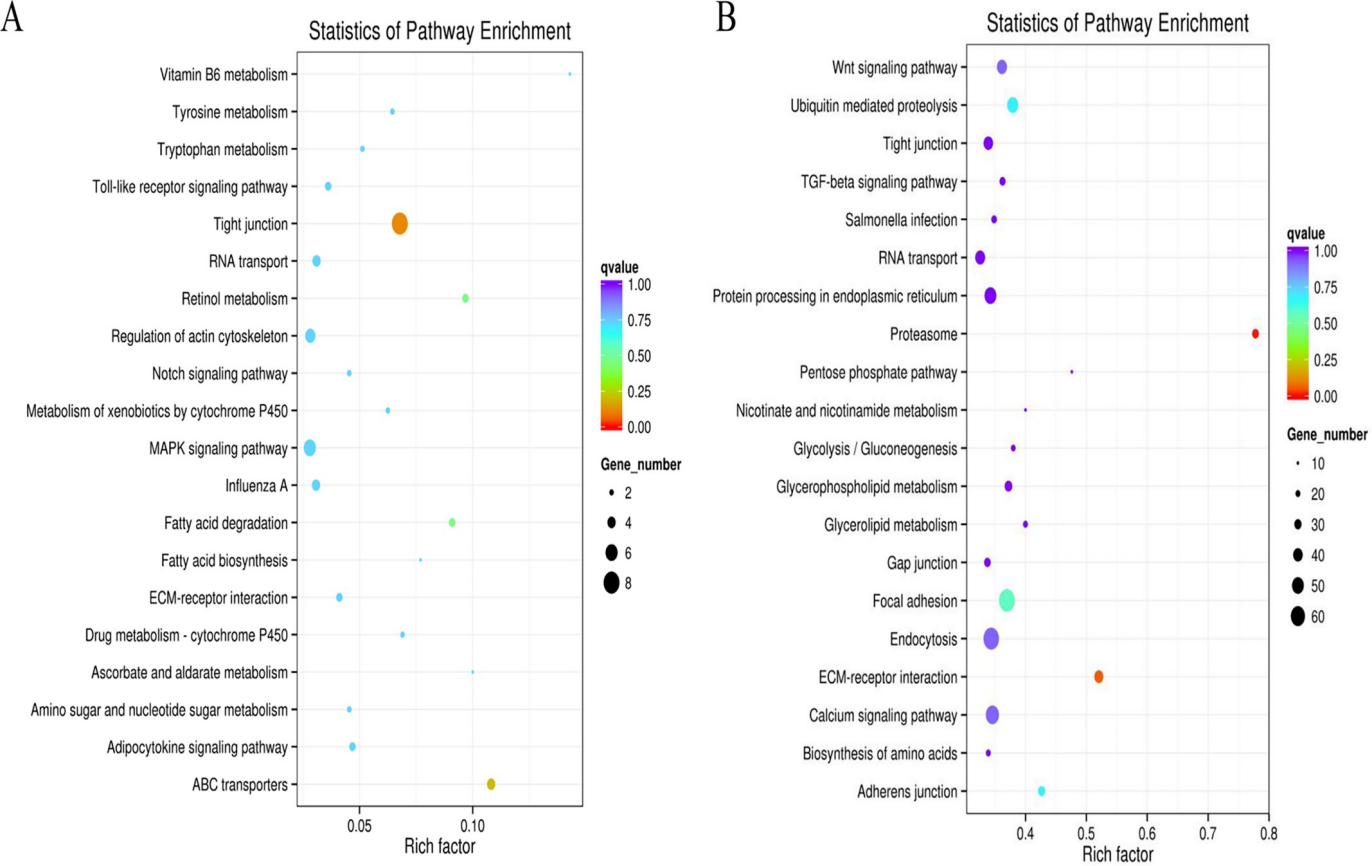
KEGG analyses of differentially expressed lncRNAs and mRNAs. (A) Scatter plot of the top 20 pathways enriched for differentially expressed lncRNAs and mRNAs in breast tissue from G20W and G55W chickens (*cis*). The abscissa represents the richness factor, and the ordinate represents the enriched pathway terms. The Q-value represents the corrected *P* (B) Scatter plot of the top 20 pathways enriched for differentially expressed lncRNAs and mRNAs in breast tissue from G20W and G55W chickens (*trans*). The abscissa represents the richness factor, and the ordinate represents the enriched pathway terms. The Q-value represents the corrected *P*.

We enriched the differentially coexpressed lncRNAs and mRNAs in the G20W and G55W groups through KEGG pathway analysis to further identify the biological pathways in which these genes participate. The pathways of the DE lncRNA target genes were significantly enriched for tight junctions, ABC transporters, retinol metabolism, fatty acid degradation and MAPK signaling pathways in the *cis* targets of lncRNAs and mRNAs (Fig 5A). For *trans* targets of lncRNAs and mRNAs, the proteasome, ECM-receptor interaction, focal adhesion, adherens junctions, ubiquitin-mediated proteolysis, endocytosis, the Wnt-signaling pathway and the calcium-signaling pathway were identified (Fig 5B). We observed that several genes were involved in skeletal muscle development and lipid metabolism, such as *COL6A3*, *COL4A1*, *COL5A1*, *COL4A2*, *LAMA4*, *LAMB4*, *LAMC1*, *Lpin1*, *PGK1*, *UBE2B*, *UBE2Q2*, and *UBE2D1* (S3 Table). Some of these pathways may be associated with muscle development and meat quality.

### LncRNA-mRNA network analysis

**Fig 6:**
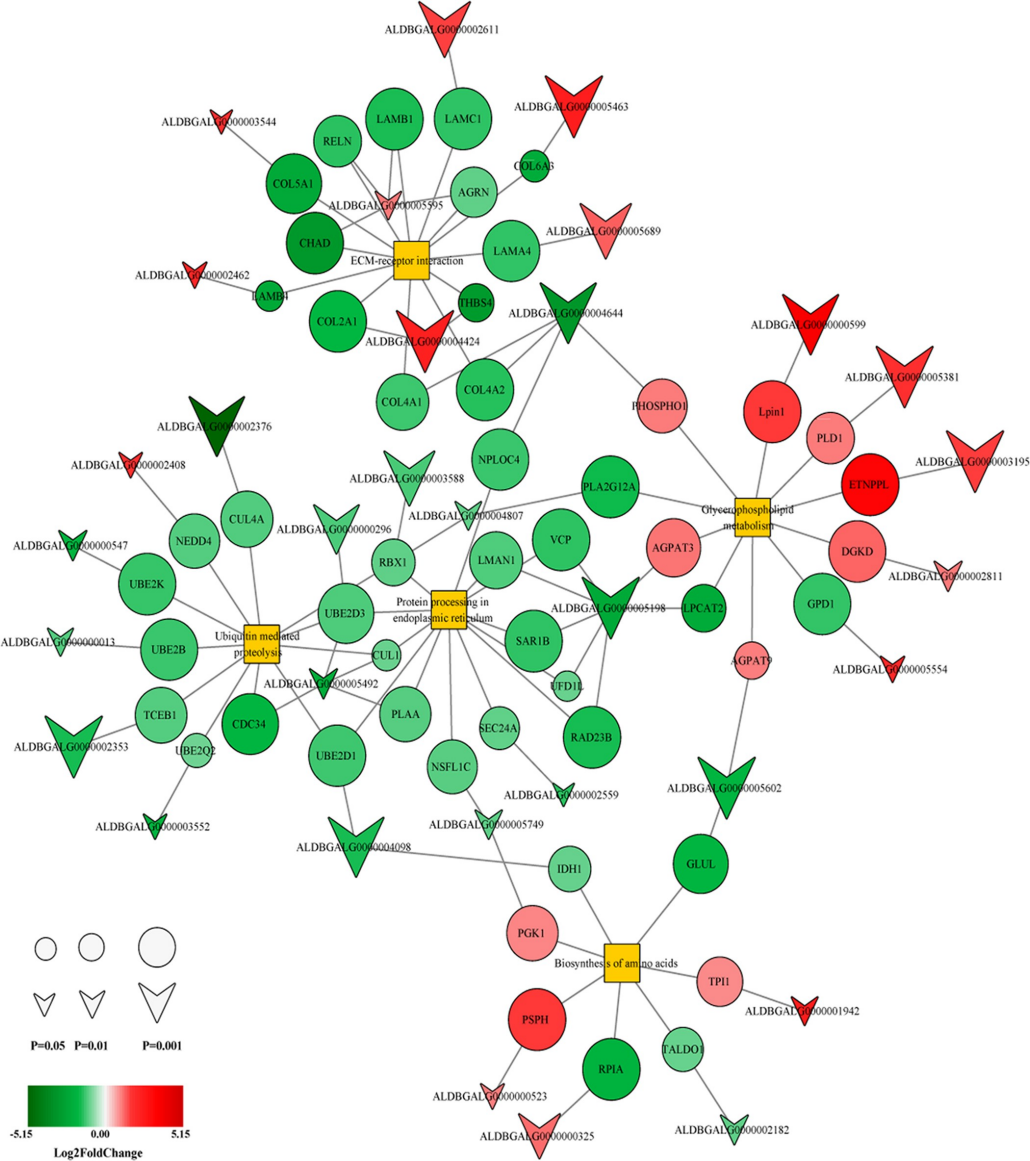
Differentially expressed lncRNA-mRNA interaction network analysis. Circles represent coding genes, and arrows represent lncRNAs. Red nodes represent upregulated nodes, and green nodes represent downregulated nodes. Yellow nodes represent pathway terms. The node size represents -logP; the smaller the *P* value is, the greater is the node size. Lines between lncRNA-mRNA represent interactions between them.

An lncRNA-mRNA network was constructed based on the correlation analysis between the differentially coexpressed lncRNAs and mRNAs, which were determined to be enriched in several pathways. We constructed gene coexpression networks to identify interactions among DE mRNAs and lncRNAs (Fig 6). The majority of DE lncRNAs and target genes (G55W vs G20W) were identified in glycerophospholipid metabolism, ECM-receptor interactions, and amino acid pathway biosynthesis. Interestingly, the expression of *PGK1*, *AGPAT9*, and *PHOSPHO1* was upregulated, and their corresponding lncRNAs, *ALDBGALG000000005749*, *ALDBGALG0000005602*, and *ALDBGALG0000004644*, respectively, were downregulated. *COL2A1*, *COL6A3*, *COL5A1*, *LAMB1*, *LAMB4*, and *LAMA4* were downregulated, and their corresponding lncRNAs were upregulated.

### Verification of lncRNA expression profiles using qRT-PCR

**Fig 7:**
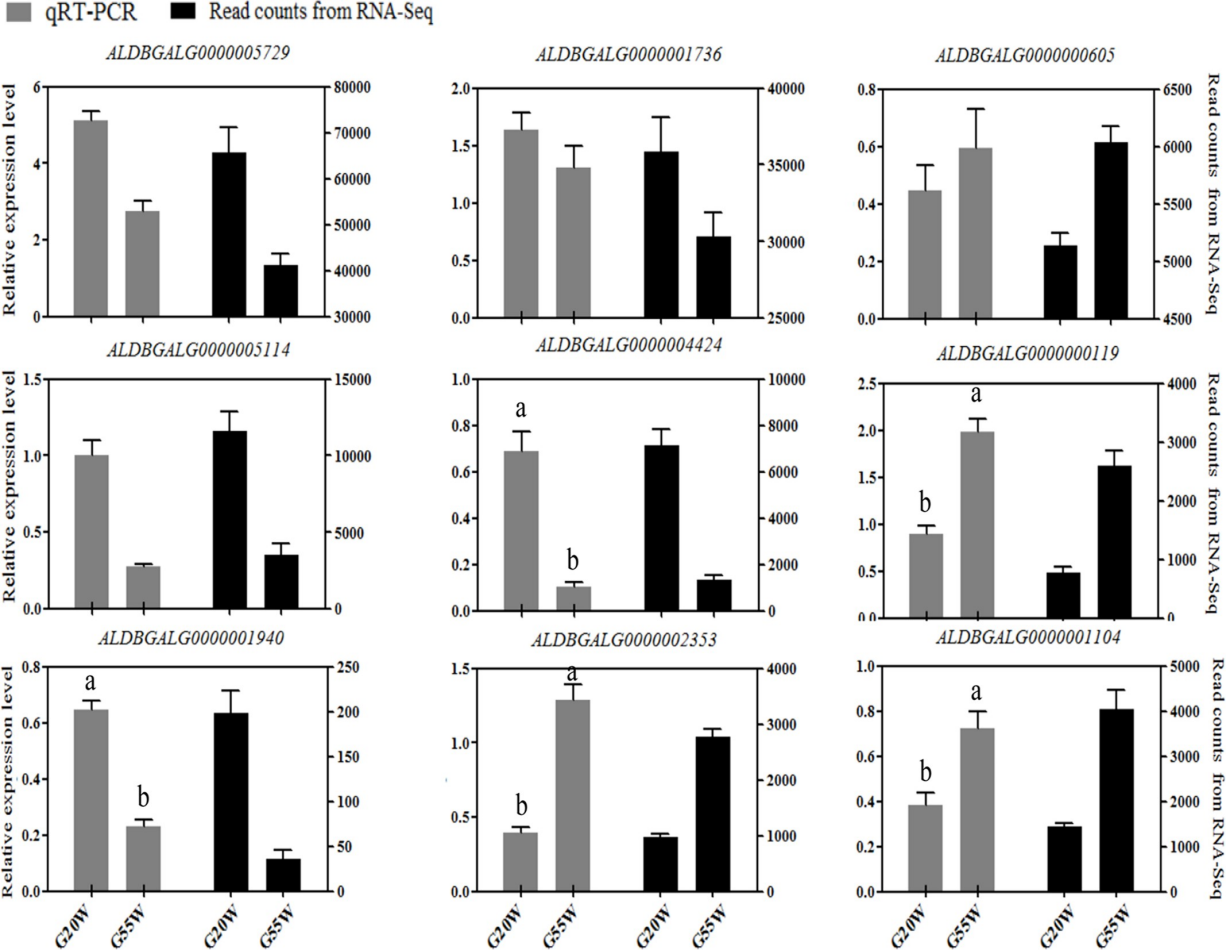
Validation of lncRNAs using RT-qPCR. Data were analyzed by the 2^-ΔΔCt^ method using *β-actin* as a reference gene. Each column represents the mean ±SE. Different letters indicate significant differences in expression levels between the two stages (*P* ≤ 0.05). Black bars represent read from RNA-Seq. Gray bars represent the results of qRT-PCR.

To verify the accuracy of our RNA-seq results, we randomly selected 9 candidate lncRNAs from common DEGs in G20W and G55W. Total RNA was obtained by the same method as for RNA-seq, and *β-actin* was used as a reference gene. The primer sequences are presented in S4 Table. The results showed that the expression of *ALDBGALG* (*5114*, *1940*, *5729*, *1736* and *4424*) was higher at 20 weeks than at 55 weeks, whereas the expression of *ALDBGALG* (*2353*, *0605*, *0119* and *1104*) was much higher at 55 weeks than at 20 weeks. All the lncRNAs expression levels were consistent with the RNA-Seq findings (Fig 7), suggesting that the detection and expression abundance of lncRNAs in our RNA-seq was highly accurate.

## Discussion

Meat quality traits are comprehensive economic characteristics controlled by polygenic traits, which are easily affected by many factors, such as castration, gender, age, and species. Species, as a major genetic factor, plays the leading role in defining the characteristics of meat quality. IMF is one of the important factors influencing meat quality and is mainly distributed in muscle and muscle fibers. IMF contains many phospholipids, and phospholipids containing large amounts of polyunsaturated fatty acids can be produced via lipid degradation reactions. IMF deposition can loosen muscle fibers, fat and connective tissue in the overlapping structure, thereby decreasing physical strength and promoting the separation of muscle fiber bundles, which especially improves the tenderness of meat [41, 42].

LncRNAs are widely distributed among mammals. Their expression is spatiotemporally and tissue specific and plays important roles in many aspects, including epigenetic regulation, transcriptional regulation and posttranscriptional regulation [43]. With the rapid development of next generation sequencing technologies, lncRNAs have been examined as novel regulatory players in cellular and biological processes using transcriptome analyses [44–49]. Some previous studies have investigated lncRNA in the muscle tissue of chickens [50, 51]. Even so, the diversity of lncRNA expression and the biological lncRNA functions at two different chicken physiological stages remain unclear. To the best of our knowledge, the lncRNA profiles in breast tissue from juvenile and laying-stage chickens are not well known. In this study, we comprehensively investigated lncRNAs in breast tissue from Gushi chickens at two physiological stages, juvenile and laying.

We used RNA-Seq technology to sequence the Gushi chicken breast tissue and identified approximately 4, 404 putative noncoding lncRNAs (4, 026 in G20W and 3, 452 in G55W). Among these lncRNAs, 131 were upregulated and 55 were downregulated in G20W compared with G55W. One difficulty in lncRNA study is that, in contrast to mRNA functions, the functions of most lncRNAs have not been determined, and there is no existing database that can be used to identify their functional annotations. Increasing evidence suggests that lncRNAs can regulate the expression of neighboring mRNAs and that their expression is highly correlated with that expression of neighboring mRNAs [9, 52]. Thus, we searched for *trans* target genes of lncRNAs to help predict the function of the lncRNAs.

The regulation of muscle development and composition is likely a function of complex networks. Therefore, examining the regulatory network is the preferred method of analysis [53]. In the present study, we constructed a coexpression network of lncRNAs and coding gene transcripts to predict the potential biological functions of DE lncRNAs. DE lncRNAs and mRNAs were enriched in several pathways in common, including protein processing in endoplasmic reticulum, ECM-receptor interaction, glycerophospholipid metabolism, ubiquitin-mediated proteolysis and the biosynthesis of amino acids, which play crucial roles in muscle development, glycerophospholipid metabolism, protein catabolism, energy metabolism and lipid metabolism processes.

The deposition of IMF is affected by a variety of genetic factors, and changes in the IMF content are closely related to different breeds and developmental stages [54]. A previous study demonstrated that ECM-receptor interaction might form a network with pathways related to lipid metabolism to influence the deposition of IMF [55]. The extracellular matrix (ECM) is a part of the connective tissue layers surrounding muscle fibers [56] and is a dynamic network structure composed of fibrous and nonfibrous proteins including collagen, proteoglycans and glycoproteins and other large molecules. The ECM provides support for the survival and activity of cells, and its effects include signal transduction that affects cell shape, function, metabolism, migration, proliferation and differentiation. Overall, there might exist a relationship between the expression of genes related to encoding collagen and ECM and meat quality traits [57]. Our results suggest that some DE genes encode collagen and epidermal growth factor-like domains, including collagen type VI alpha 3 chain (*COL6A3*), collagen type IV alpha 1 chain (*COL4A1*), collagen type V alpha 1 chain (*COL5A1*), collagen type IV alpha 2 chain (*COL4A2*), collagen type II alpha 1 chain (*COL2A1*), laminin subunit alpha 4 (*LAMA4*), laminin subunit beta 4 (*LAMB4*), laminin subunit gamma 1 (*LAMC1*), laminin subunit beta 1 (*LAMB1*), and reelin (*RELN*). It was suggested that these genes have an important relationship to the ECM [58]. Among them, *LAMA4*, which is encoded by the *LAMA4* gene, mainly functions outside neuronal membranes, in muscle fibers during the development of endothelial cells and surrounding the basement membrane. Another study suggested that *LAMA4* likely functions as a key protein in muscle development during the embryonic period. In our study, *COL2A1*, *COL6A3*, *COL5A1*, *LAMB1*, *LAMB4*, and *LAMA4* were downregulated, and their corresponding DE lncRNAs (*ALDBGALG 2611*, *5463*, *5689*, *3544*, *4424* and *5595*) were upregulated. The upregulation of a given lncRNA may inhibit its target gene and affect meat quality, a possibility that requires further study.

Lipid phosphate phosphohydrolase 1 (*Lpin1*) is a member of the LPIN family and is a key gene in adipocyte differentiation and lipid metabolism. In a previous study using RNA-Seq in broilers of two groups with different shear forces of breast tissue, the *LPIN1* gene was found to be downregulated and involved in the regulation of meat tenderness [59]. The corresponding lncRNA, *ALDBGALG599*, may have a similar function, and both may influence meat quality. Interestingly, we found that *PGK1* (phosphoglycerate kinase 1), a key glycolytic enzyme involved in the biosynthesis of amino acids, was upregulated at two different chicken physiological stages. A lack of this enzyme in organisms can cause metabolic disorders. Another study suggested that *PGK1* was involved in acid synthesis and influenced pig IMF deposition in skeletal muscle, affecting the flavor of meat. In our study, the corresponding lncRNA of *PGK1*, *ALDBGALG5749* was downregulated, and its function in chickens should be examined in future studies.

Ubiquitin-mediated proteolysis may play a crucial role in protein degradation during the aging process. Protein degradation also affects muscle shear and thus alters meat tenderness [2, 60, 61]. Our results suggest that some differentially coexpressed genes are associated with ubiquitin modification, including ubiquitin-conjugating enzyme E2 B (*UBE2B*), ubiquitin conjugating enzyme E2 Q2 (*UBE2Q2*), ubiquitin conjugating enzyme E2 D1 (*UBE2D1*), ubiquitin conjugating enzyme E2 D3(*UBE2D3*) and ubiquitin conjugating enzyme E2 K (*UBE2K*). The results indicate that co-expressed genes might affect the expression of these ubiquitin-related genes and thus participate in protein degradation in breast muscle.

## Conclusion

In conclusion, we first generated the expression profiles of lncRNAs in breast tissue from two developmental stages of Gushi chicken (G20W and G55W) based on RNA-Seq. Our results suggest that those lncRNAs might play important roles in meat quality. Overall, this study takes the first step toward understanding the molecular mechanisms underlying variations in poultry meat quality and helps to provide a theoretical basis for further research. However, the regulatory mechanisms of lncRNAs require further verification.

## Supporting information

S1 Table Cis target genes of lnc RNAs. (XLSX)

S2 Table Trans target genes of lncRNAs. (XLSX)

S3 Table KEGG pathways associated with lncRNA-mRNA interaction network analysis of differentially expressed genes. (XLSX)

S4 Table Primers used for qRT-PCR in this study. (XLSX)
